# Editorial: The role of inflammasome in viral infection, volume II

**DOI:** 10.3389/fcimb.2024.1438310

**Published:** 2024-06-12

**Authors:** Pin Wan, Pan Pan, Binlian Sun, Yongkui Li

**Affiliations:** ^1^ Hubei Key Laboratory of Cognitive and Affective Disorders, Institute of Biomedical Sciences, School of Medicine, Jianghan University, Wuhan, China; ^2^ School of Basic Medical Sciences, Guangzhou Medical University, Guangzhou, China; ^3^ Laboratory of Viral Pathogenesis & Infection Prevention and Control, Jinan University, Ministry of Education, Guangzhou, China; ^4^ Institute of Medical Microbiology, Department of Immunology and Microbiology, College of Life Science and Technology, Jinan University, Guangzhou, China

**Keywords:** inflammasome, RNA viruses, SARS-CoV-2, influenza A virus, chronic viral hepatitis

The Inflammasome, belonging to the pattern recognition receptors (PRRs) family, plays indispensable roles in pathogen infections (Chen and Sun, 2013). It can be stimulated by pathogen-associated molecular patterns (PAMPs) or damage-associated molecular patterns (DAMPs), including ATP, monosodium urate (MSU), silica crystals, fibrillar amyloid-β peptide, aluminum salt crystals, many different kinds of RNA Viruses, and fungi (Chen and Sun, 2013; He et al., 2016). Activation of the inflammasome leads to maturation and secretion of pro-inflammatory cytokines interleukin-1β (IL-1β) and IL-18, as well as cleavage of gasdermin D (GSDMD) that mediates pyroptosis (Xu and Nunez, 2022). In recent years, some studies revealed that many kinds of viruses could regulate the inflammasome by direct mechanisms (viral proteins or viral nucleic acids could regulate the activation of the inflammasome) or indirect pathways (viruses could affect the activation of NLRP3 by shaping and modulating other signal pathways or other proteins). Previous studies have indicated that the inflammasome participates in viral infectious diseases (Xu and Nunez, 2022; Hadad et al., 2023). However, what role the inflammasome plays in viral infections needs more specific research.

In this Research Topic, there are four articles (two original research articles and two review articles) published, focusing on the role of the inflammasome in viral infection.

Coronavirus disease 2019 (COVID-19) was caused by the infection of severe acute respiratory syndrome coronavirus 2 (SARS-CoV-2), which activates the NLRP3 inflammasome (Pan et al., 2021; Hadad et al., 2023). Liu et al. found a rising reinfection risk of emerging immune evasion variants like omicron JN.1 in China, and suggested the importance of boosting with updated vaccines. They tracked a 2-year-long cohort who had received three doses of inactivated COVID-19 vaccine, and found that the constant mini-waves of SARS-CoV-2 infections caused by the emerging variants were related with drastic immune evasion and decreasing sera neutralization of convalescents along time.

Influenza A virus (IAV) infection causes respiratory diseases in humans and other animals, and highly pathogenic IAV caused pandemics, leading to high mortality (Chung et al., 2015). IAV activates the NLRP3 inflammasome via its NS1 protein or PB1-F2 protein (Chung et al., 2015; Sarvestani and McAuley, 2017; Cheung et al., 2020). Huang et al. elucidated the multifaceted interplay of patient factors, innate immunity, and inflammasome responses in lung tissues subjected to ex vivo H3N2 IAV exposure, reflecting real-world lower respiratory tract infections. They demonstrated the minute responses of human lung tissue to *ex vivo* H3N2 IAV infections, underscoring the central role of NP-expressing macrophages in the formative stages of the immune response. Histories of lung cancer, aging, and previous encounters with IAV emerge as critical factors that shape and modulate these responses.

Viral hepatitis triggers a wide array of liver diseases and causes significant damage to human health and life safety (Odenwald and Paul, 2022). Wan et al. systemically summarized the concrete content of different types of inflammasomes (including NLRP3, NLRC4, IFI16 and AIM2) in HBV/HCV infection and related diseases. They presented that HBV/HCV activates NLRP3 inflammasome by viral proteins-mediated assembly of the inflammasome components and viral proteins/RNA-mediated activation of NF-kB signaling pathways. Collectively, they showed that the complex interplay between chronic viral hepatitis and inflammasome activation enhances the understanding of the pathological mechanisms of chronic hepatitis and hepatocellular carcinoma.

RNA viruses, characterized by their RNA genomes, are tremendous threats to human life and health (Garcia-Blanco et al., 2022; Mittelholzer and Klimkait, 2022). Yue et al. summarized the detailed regulation of the NLRP3 inflammasome in positive-sense single-stranded RNA virus infection, the effects of the NLRP3 inflammasome activation in negative-sense single-stranded RNA virus, the role of the NLRP3 inflammasome activation in double-stranded RNA virus, the clinical significance of NLRP3 inflammasome activation in viral diseases, and therapeutic strategies targeting the NLRP3 inflammasome for anti-inflammatory effects in RNA virus infection. They specially emphasized that the activation of the NLRP3 inflammasome was regulated by various encoding proteins of RNA viruses, viral RNA, and NF-kB signaling pathway/ROS generation/potassium efflux induced by viral proteins or RNA. They explained the roles of the NLRP3 inflammasome in infection of RNA viruses including SARS-CoV, SARS-CoV-2, Dengue virus (DENV), Zika virus (ZIKV), Enterovirus 71 (EV71), Human Immunodeficiency Virus (HIV), Influenza virus, Ebola virus (EBOV), Respiratory Syncytial virus (RSV), Rift Valley Fever virus (RVFV), Hantavirus (HTNV) and Reovirus. They also summarized the developed small-molecule drugs targeting the NLRP3 inflammasome including Cannabidiol, Statins, Berberine, probenecid, AZ11645373, PD098059, Caffeic acid phenethyl ester (CAPE), Disulfiram (DSF), MCC950 and CY-09. Collectively, they showed the complex interplay between RNA virus infections and the NLRP3 inflammasome, and also showed various approaches to suppress the activation of the NLRP3 inflammasome by inhibiting NF-kB signaling pathway, NLRP3 inflammasome assembly, and GSDMD pore formation.

These papers collected by this Research Topic show both new findings and detailed summaries of studies within the notion of this topic. Notably, there are still many scientific questions about the role of inflammasome in viral infection. For example, what is the key physiological inducer of inflammasome overactivation in severe infections *in vivo*, how the negative regulatory pathways of the inflammasome act in viral infections, and so on, are worth to be studied in the future. Revealing the role of the inflammasome in viral infections will help understanding the pathogenesis of viral infectious diseases.

## Author contributions

YL: Writing – review & editing. PW: Writing – original draft. PP: Writing – original draft. BS: Writing – review & editing.
